# AI-based CT assessment of 3117 vertebrae reveals significant sex-specific vertebral height differences

**DOI:** 10.1038/s41598-025-05091-0

**Published:** 2025-07-01

**Authors:** Viktoria Palm, Subasini Thangamani, Bettina Katalin Budai, Stephan Skornitzke, Kira Eckl, Elizabeth Tong, Sam Sedaghat, Claus Peter Heußel, Oyunbileg von Stackelberg, Sandy Engelhardt, Taisiya Kopytova, Tobias Norajitra, Klaus H. Maier-Hein, Hans-Ulrich Kauczor, Mark Oliver Wielpütz

**Affiliations:** 1 Clinic for Diagnostic and Interventional Radiology (DIR), Heidelberg University Hospital, Heidelberg, Germany; 2 Diagnostic and Interventional Radiology with Nuclear Medicine, Thoraxklinik Heidelberg, Heidelberg, Germany; 3 Philips GmbH Market DACH, Hamburg, Germany; 4 Department of Internal Medicine III, University Hospital Heidelberg, Heidelberg, Germany; 5 DZHK (German Centre for Cardiovascular Research), partnersite Heidelberg/Mannheim, Heidelberg, Germany; 6 Department of Radiology, Netherlands Cancer Institute-Antoni van Leeuwenhoek Hospital, Amsterdam, The Netherlands; 7 Division of Medical Image Computing, German Cancer Research Center, Heidelberg, Germany; 8 Pattern Analysis and Learning Group, Department of Radiation Oncology, Heidelberg University Hospital, Heidelberg, Germany; 9 Department of Diagnostic Radiology and Neuroradiology, University Medicine Greifswald, Ferdinand-Sauerbruch-Strasse 1, 17475 Greifswald, Germany; 10 Clinic for Nuclear Medicine, University Medicine Greifswald, Ferdinand- Sauerbruch-Strasse 1, 17475 Greifswald, Germany

**Keywords:** Spine, Sex characteristics, Image interpretation, computer-assisted, Anthropometry, Artificial intelligence, Image processing, computer-assisted, Bone, Translational research

## Abstract

Predicting vertebral height is complex due to individual factors. AI-based medical imaging analysis offers new opportunities for vertebral assessment. Thereby, these novel methods may contribute to sex-adapted nomograms and vertebral height prediction models, aiding in diagnosing spinal conditions like compression fractures and supporting individualized, sex-specific medicine. In this study an AI-based CT-imaging spine analysis of 262 subjects (mean age 32.36 years, range 20–54 years) was conducted, including a total of 3117 vertebrae, to assess sex-associated anatomical variations. Automated segmentations provided anterior, central, and posterior vertebral heights. Regression analysis with a cubic spline linear mixed-effects model was adapted to age, sex, and spinal segments. Measurement reliability was confirmed by two readers with an intraclass correlation coefficient (ICC) of 0.94–0.98. Female vertebral heights were consistently smaller than males (*p* < 0.05). The largest differences were found in the upper thoracic spine (T1–T6), with mean differences of 7.9–9.0%. Specifically, T1 and T2 showed differences of 8.6% and 9.0%, respectively. The strongest height increase between consecutive vertebrae was observed from T9 to L1 (mean slope of 1.46; 6.63% for females and 1.53; 6.48% for males). This study highlights significant sex-based differences in vertebral heights, resulting in sex-adapted nomograms that can enhance diagnostic accuracy and support individualized patient assessments.

**Supplementary Information:**

The online version contains supplementary material available at 10.1038/s41598-025-05091-0.

## Introduction

Twenty-four distinct vertebrae of the human spine, each defined by a specific gross anatomy and height, serve individual biodynamic requirements^1^. Vertebral height distribution in humans has been studied in the context of spinal morphology, pathology, and biomechanics^2–4^. In forensic body height estimation, regression models have been developed based on specific anatomical regions utilized in forensic anthropology^5–9^. These models rely on the principle that a proportional relationship exists between certain anatomical features of the spine and the individual’s height. Therefore, data from anthropometric studies were evaluated by population-based regression analyses of mean heights to estimate the stature of the deceased^1,10–14^. Moreover, spinal regression techniques in medical imaging are commonly employed for spinal morphometry and disease assessment, such as assessing scoliosis or vertebral compression fractures in 200 million osteoporosis patients worldwide^15^. Based on vertebral wedge indices, Genant et al. developed a semiquantitative, osteoporotic fracture grading system based on vertebral height reduction, which is still the gold standard for radiological assessment of osteoporotic fractures^16^.

Medical imaging has been utilized to analyze vertebral body heights and dimensions in healthy individuals^17–20^. First efforts to define normal vertebral dimensions in medical imaging have been made in 1991 by Dennis M. Black et al.^21^. Anterior and posterior vertebral heights and their corresponding relations were evaluated based on measurements in radiographs. Later, research based on manual measurements of vertebral height in computed tomography (CT) imaging showed likewise feasibility with increased anatomical accuracy^1^. Regression-based analyses leverage on cross sectional imaging modalities to accurately measure spinal structures enabling a comprehensive evaluation for spinal disorder management, preoperative planning, and forensic identification. However, manual measurement techniques were inherently limited by inter- and intra-observer variability leading to the demand for more standardized and reproducible imaging-based approaches^22^.

Population-based studies on vertebral height distribution and confirmed sex-based differences, with men generally exhibiting larger vertebrae than women^23,24^. While these studies provided fundamental insights into sex-related vertebral morphology, longitudinal changes in vertebral height based on the impact of increasing human stature over generations have not been assessed. Hence, given that the stature of the human population increases each decade from 1 to 3 cm, previously established reference values and vertebral height models may be outdated^25^.

Modern image-analysis approaches allow automation of the height correlation of neighboring vertebrae, enabling research for standardized large-scale studies to provide reproducible height measurements and new sex-based vertebral height models.

Our study addresses this gap by combining large-scale automated vertebral height extraction from CT for sex-adapted nomograms and providing updated vertebral height reference values, both may contribute for personalized osteoporosis risk-stratification in future. Applying the results of our algorithm based anatomical analysis of 3117 vertebrae, we developed an improved, sex-related regression model as well as a Pseudo-Jacobian matrix, as analytical framework for intra-individual vertebral height comparisons, addressing the complex anatomy of the thoracolumbar spine. By integrating CT analysis with new sex-specific vertebral height models, our study is expected to reference for future applications in osteoporosis-related fracture detection and personalized risk assessment.

## Materials and methods

### Patient selection

This retrospective study was approved by the institutional review board Heidelberg University, Ethics Committee of the Medical Faculty, Heidelberg, Germany under number S-937/2020. The study was performed in accordance with the Declaration of Helsinki 2013. Informed consent was waived by the institutional review board Heidelberg University, Ethics Committee of the Medical Faculty, Heidelberg, Germany, and all methods were carried out in accordance with state-of-the-art guidelines and regulations.

The CT study dataset was retrospectively and sequentially enrolled from the PACS systems from the Thoraxklinik Heidelberg and Heidelberg University Hospital. Patients who underwent routine spine, chest and/or abdominal CT examinations between January 2016 and January 2021 were manually selected. No randomization was applied, as all eligible datasets were included to ensure a comprehensive assessment of visually healthy thoracolumbar spines. Image reviewing was conducted systematically by an experienced, board-certified radiologist (> 6 years of experience) and two specifically-trained Ph.D. students (> 1 year of experience). Additionally, all decisions from the Ph.D. students were overseen by a senior radiologist to maintain accuracy. The inclusion of observers with varying experience ensured both expert verification and efficient data review. The cervical spine was excluded due to its highly varying anatomy compared to the thoracolumbar spine. To minimize selection bias, all datasets meeting the predefined inclusion criteria were considered without preference for age, or sex distribution.

**Fig. 1:**
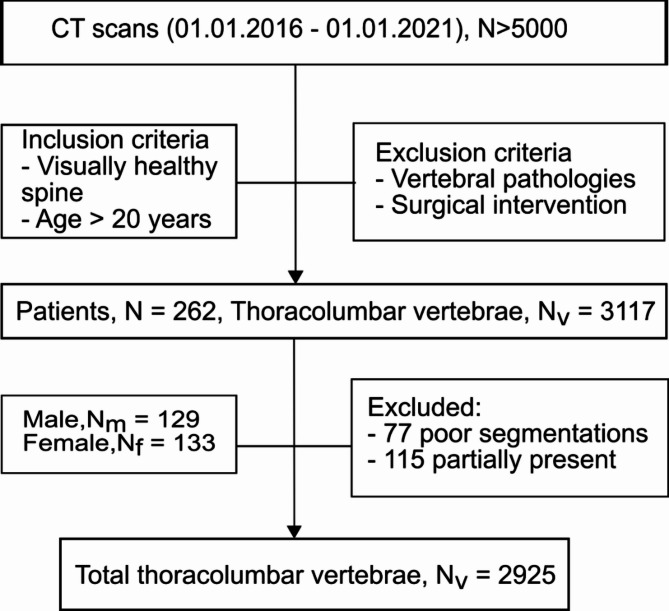
Inclusion and exclusion criteria flowchart of the study cohort.

Anatomical aberrations concerning shape/height/volume of the thoracolumbar spine (e.g. butterfly vertebrae, vertebrae plana, hemangioma), previous medical spine interventions, and vertebral pathologies (e.g. significant degenerations, metastasis, fractures) were strictly excluded, resulting in a study cohort of 262 subjects, including a total of 3117 vertebrae (Fig. 1).

Image acquisition included 2 manufacturers with overall 5 different CT scanner types:

- A Dual-source 128-slice scanner (SOMATOM Definition Flash); Dual-source 256-slice scanner (SOMATOM Force); 16-detector-row scanner (Emotion 16); 64-detector-row scanner (SOMATOM Definition AS) from Siemens Healthineers (Erlangen, Germany).
- A Spectral CT scanner with dual-layer detector (IQon) from Philips Healthcare (Amsterdam, Netherlands).

Image acquisition parameters and imaging protocol varied based on the manufacturer, CT scanner type, and individual factors, such as BMI and the primary diagnostic request for the CT examination. Hence, several image acquisition parameters were included, such as tube voltage 70–140 kVp, various contrast phases, and reconstruction kernels. Anonymized image export was done automatically with an in-house developed software (ADiT, Automated DICOM Transfer)^26^.

### Data collection

#### Vertebral height extraction

##### Segmentation

**Fig. 2:**
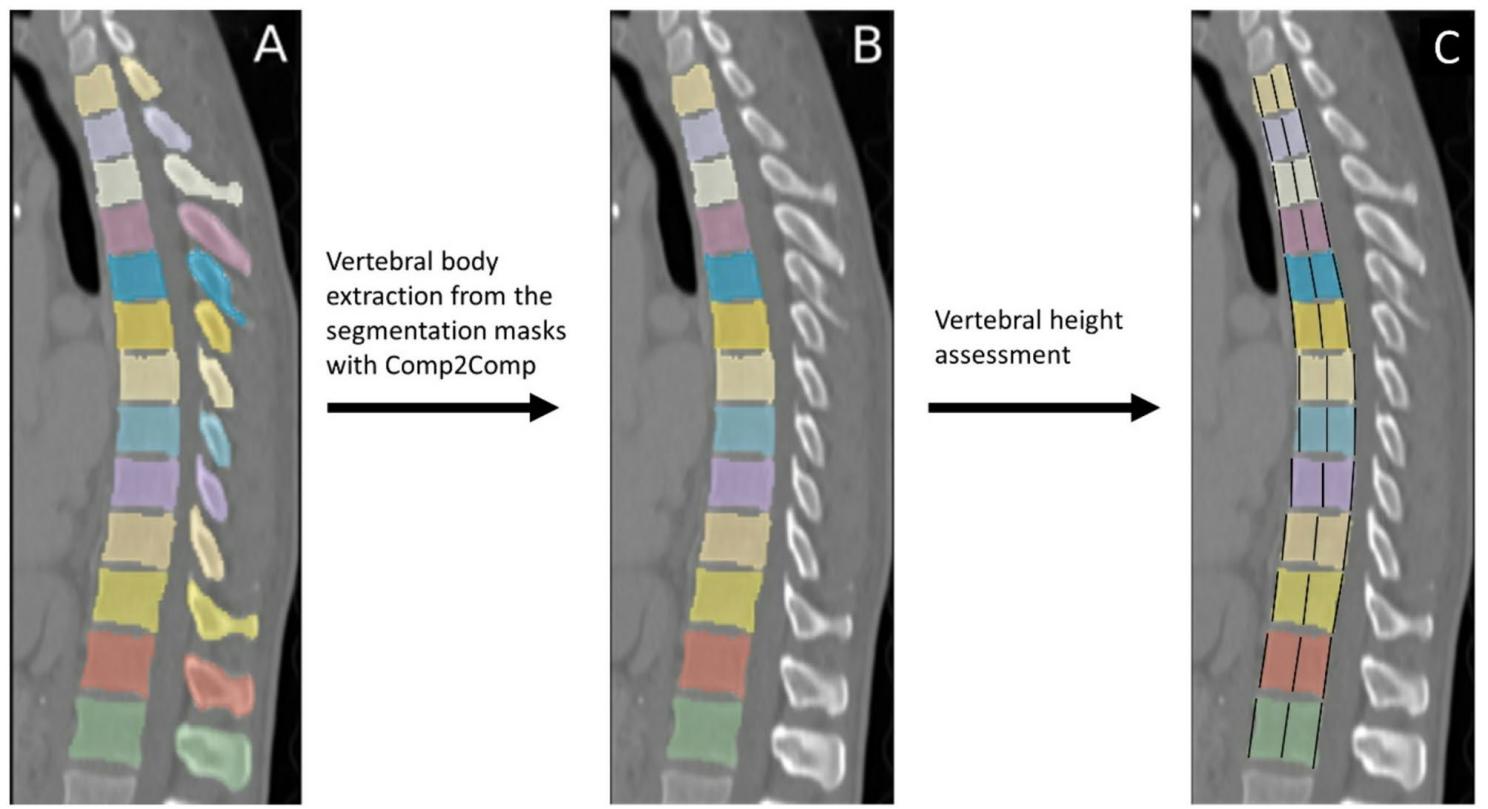
(**A**) Segmentations of vertebrae from T1 to L1 from TotalSegmentator. (**B**) Vertebral body extraction with Comp2Comp. (**C**) Assessment of vertebral height.

The CT images were manually cropped using 3D Slicer (v5.4.0) to remove any background besides the spine. The anatomical segmentation of thoracic and lumbar vertebrae was performed using the deep learning-based algorithm TotalSegmentator (Fig. 2A)^27^. This open source algorithm has been validated in previous studies, demonstrating high segmentation accuracy with a mean Dice similarity coefficient (DSC) of ≥ 0.95 and a Jaccard index exceeding 0.90, confirming its robustness and reliability^27^. The segmentations comprised the whole vertebra including the vertebral body, pedicles, spinous process and articular structures (Fig. 2A).

77 misssegmentations and 115 partially-displayed vertebrae were manually excluded (Fig. 1). Extraction of the vertebral centroid sagittal 2D slice at the level of the spinous process was done by automated detection of the two largest objects (vertebral body, spinous process) in the 3D segmentation mask. Only the vertebral body was required for the intended analysis. The removal of the posterior processes from the obtained 2D sagittal image mask was performed by the open-source Python package Comp2Comp (Fig. 2B)^28^.

##### Vertebral height assessment

**Fig. 3:**
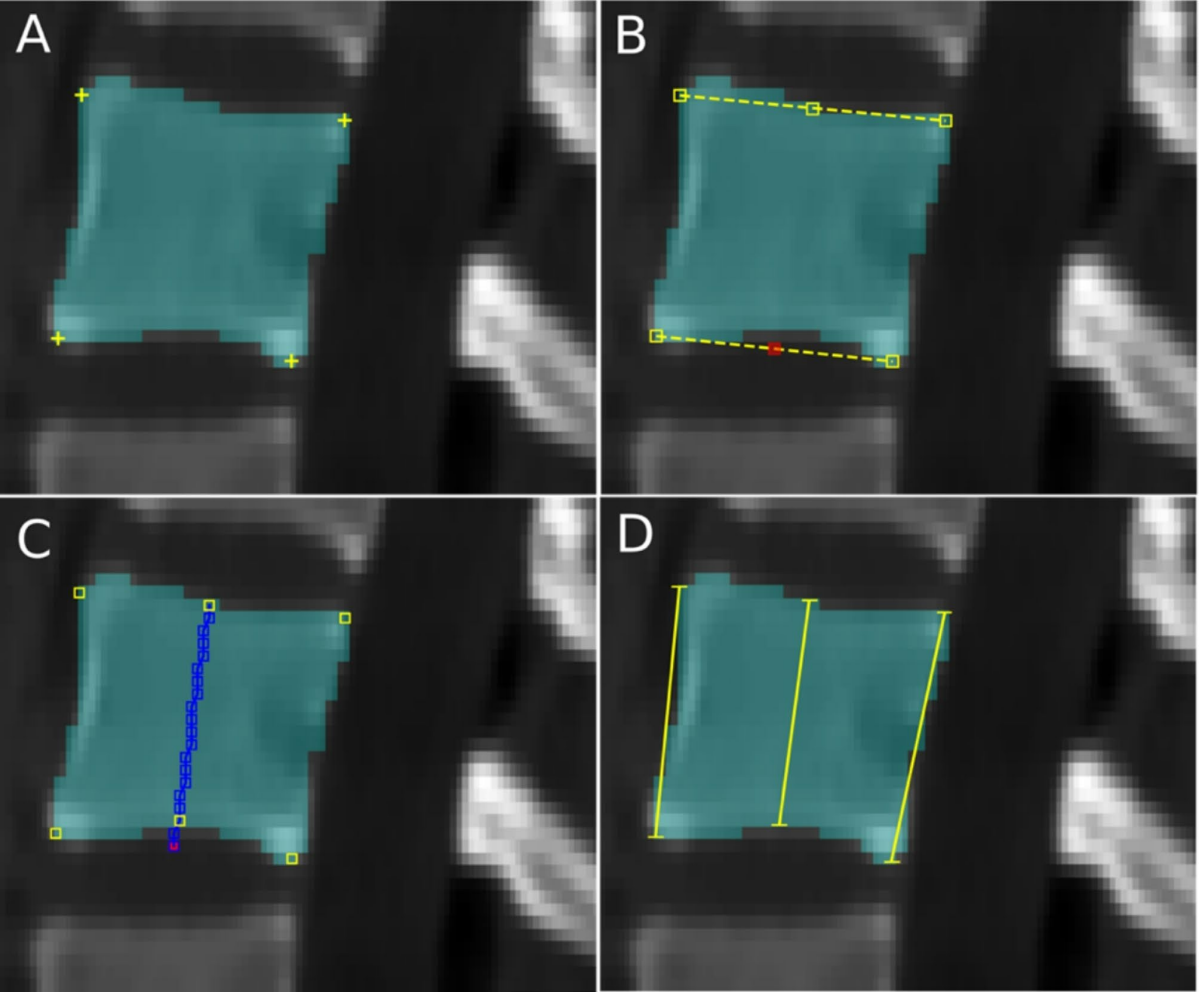
Assessment of vertebral heights. (**A**) Yellow crosses indicate the identified corner pixels. (**B**) Midpoints may lie on background pixels (red square). (**C**) Line drawing algorithm (blue line) in between the midpoints gives the most outer pixels on the segmentation mask (yellow rectangles). (**D**) Euclidean distance measurement for H_a_, H_p_, and H_c_ (yellow lines).

For the vertebral height assessment (Fig. 2C) the landmark points for vertebral height calculation were obtained through the following two steps using the OpenCV python package:

1. Binary thresholding was applied to the 2D slice such that all pixel values in the vertebral mask are set to either 0 or 255.
2. The contours in this 2D mask were identified and approximated to a polygonal curve with four vertices (Fig. 3A).

Anterior (H_a_) and posterior (H_p_) heights were calculated by assessing the distance of the anterior and posterior vertices. Calculating the central vertebral height (H_c_) by taking the midpoints of the distance from the anterior to posterior vertices could result in pixels on the background beyond the segmentation mask (Fig. 3B). Hence the outermost pixel on the mask at the midpoint level has been identified by the line drawing Bresenham’s algorithm (Fig. 3C)^29^. Euclidean distance between the landmark points (pixels) resulted in H_a,_ H_p_ and H_c_ (Fig. 3D).

##### Validation

22 subjects were randomly chosen from the study cohort for manual Ha, Hp and Hc assessment. The sample size was determined based on statistical guidelines for reliability analysis, where a minimum of 20–25 subjects is recommended to achieve 95% statistical power with a confidence interval of ±0.05 when assessing inter-reader reliability^30–32^. A total of 336 vertebrae were measured by an experienced radiologist and a trained, first-year Ph.D. student in the clinical PACS (Centricity, Universal Viewer, 7.0 SP1.0.1, GE Healthcare, Chicago, USA, 2023). The reliability of automated measurements was assessed based on the intraclass correlation coefficient (ICC (2, k))^33,34^. A two-way random effects model was chosen, and the absolute agreement was calculated.

### Statistical analyses

Normal distribution, mean, and standard deviation (SD) were assessed from Ha, Hp and Hc. The anterior wedge index (Ha/Hp) and the biconcavity index (Hc/Hp) were calculated for males and females individually. The statistical analysis was done in Python (3.11.5) and R (version 4.3.2). Student’s t-test was used to analyze sex differences. P < 0.05 was considered statistically significant. 

#### Mixed model regression

Two spline regression models were compared in the analysis to model the vertebral heights and perform a sex-wise comparison of the model fit. The vertebral label was used as the predictor variable and the vertebral height as the response variable. Separate models were fitted for Ha, Hp and Hc. The cubic spline regression model was chosen to take nonlinearity into account. Linear spline regression was performed to assess the fitting of a simpler model concerning vertebral height distribution. Both sex-stratified and pooled-sex analyses were performed separately. For each model, intercept, beta coefficients (β), marginal R squared values (R2 proportion of variance explained by fixed factors) and conditional R squared values (R2 proportion of variance explained by fixed and random factors) were determined.

#### Pseudo-Jacobian matrix

Evaluation of height proportions beyond neighboring vertebrae, including the intra-individual proportions of all thoracolumbar vertebrae to each other, was assessed by a Pseudo-Jacobian matrix of size N x N, where N represents the number of vertebrae. Here, the ratios of the automated height measurements have been calculated, relating every given vertebra to one another intra-individually. The corresponding mean matrices relate to the mean height ratios of the study cohort in Ha, Hp and Hc. Moreover, standard mean height and sex-dependent ratio matrices were calculated. To assess sex-associated differences in the ratios, the mean Pseudo-Jacobian matrices were plotted as three-by-three matrices referring to three regions T1-T9, T10-L1, and L2-L5, corresponding to the cubic spline analyses.

## Results

### Cohort description

The study cohort includes a total of 262 adult subjects (M = 129) with a mean age of 32.36 years (20–54 years). Visual assessment of the automated, anatomical segmentation from 3117 vertebrae revealed misssegmentations or incomplete presence in 6.16% (*N* = 192). Correspondingly, for further evaluations a total of 8775 height measurements in 2062 thoracic and 863 lumbar vertebrae were achieved (Table 1).

**Table 1:** Subject data of study cohort.

Total number of subjects	262
Sex (f/m)	133/129
Age (years)	31 ± 8
Vertebral body count	2925
Thoracic	2062
Lumbar	863

### Validation of automated height measurements

Comparison of the manual height measurements of the two readers (R1 & R2) as well as manual vs. automated measurements showed acceptable ICC values of 0.94–0.98 (Table S1). There were no significant differences in the ICC between these three evaluations with p-value ≤ 0.001, verifying that automated measurements were as reliable as manual measurements.

### Sex-differences in vertebral heights

Automated measurements of the mean H_a,_ H_p_ and H_c_ show a continuous increase in craniocaudal direction with an S-shaped distribution in males and females (Fig. S1). A height-plateau was demonstrated from T5 to T8 (mean slope 0.29; 1.64% for females and 0.23; 1.21% for males) and L2 to L5 (mean slope − 0.07; − 0.26% for females and − 0.22; − 0.83% for males), most prominent in H_a_ and H_c_. The highest increase of heights between neighboring vertebrae in craniocaudal direction was found in the lower thoracic spine from T9 to L1 in both sexes (mean slope 1.46; 6.63% for females and 1.56; 6.63% for males). The largest sex-based differences in mean heights comparing the same vertebral height of male and female were found from T1 to T6 with delta 7.9-9.0%, specifically T1 (1.34 mm; 8.6%), T2 (1.55 mm; 9.0%) (Table 2).

**Table 2:** Mean vertebral heights of males and females with vertebra-specific absolute and relative differences showing the highest delta in the upper thorax and the lowest delta in the lumbar spine.

	MALE	FEMALE	Delta	Delta
	Mean Height (mm)	Mean Height (mm)	mm	%
T1	15.69	14.35	1.34	8.6%
T2	17.11	15.56	1.55	9.0%
T3	17.44	16.01	1.43	8.2%
T4	18.28	16.83	1.45	7.9%
T5	19.14	17.61	1.53	8.0%
T6	19.49	17.89	1.60	8.2%
T7	19.38	17.91	1.47	7.6%
T8	19.22	18.01	1.21	6.3%
T9	20.47	18.89	1.57	7.7%
T10	22.13	20.36	1.77	8.0%
T11	23.24	21.76	1.48	6.4%
T12	24.94	23.63	1.31	5.3%
L1	26.84	25.30	1.55	5.8%
L2	27.26	26.20	1.06	3.9%
L3	27.62	26.36	1.25	4.5%
L4	27.13	26.03	1.09	4.0%
L5	26.13	25.03	1.10	4.2%

The smallest mean height delta between males and females was found in the lumbar spine, specifically in L2 to L5 with values < 5% (3.9–4.5%). Supplement Table S2 shows sex-based mean H_a,_ H_p,_ H_c_ and SD. On individual vertebral segments, heights showed significant differences between males and females (*p* < 0.001) except for H_a_ T12 (*p* = 0.11), L2 (*p* = 0.06) and L4 (*p* = 0.07) (Table 3). A power analysis for non-significant results achieved statistical power levels of 95–99% for detecting moderate effect sizes (Cohen’s d > 0.4). This indicates that if meaningful sex differences existed at this level, our study would have reliably detected them.

**Table 3:** Significant differences of H_a_, H_c_, and H_p_ in males and females.

	Anterior height	Central height	Posterior height
	P-value	P-value	P-value
T1	< 0.001	< 0.001	< 0.001
T2	< 0.001	< 0.001	< 0.001
T3	< 0.001	< 0.001	< 0.001
T4	< 0.001	< 0.001	< 0.001
T5	< 0.001	< 0.001	< 0.001
T6	< 0.001	< 0.001	< 0.001
T7	< 0.001	< 0.001	< 0.001
T8	< 0.001	< 0.001	< 0.001
T9	< 0.001	< 0.001	< 0.001
T10	< 0.001	< 0.001	< 0.001
T11	< 0.001	< 0.001	< 0.001
T12	0.11	< 0.001	< 0.001
L1	< 0.001	< 0.001	< 0.001
L2	0.06	< 0.001	< 0.001
L3	< 0.001	0.001	< 0.001
L4	0.07	< 0.001	< 0.001
L5	< 0.001	0.003	0.02

**Fig. 4:**
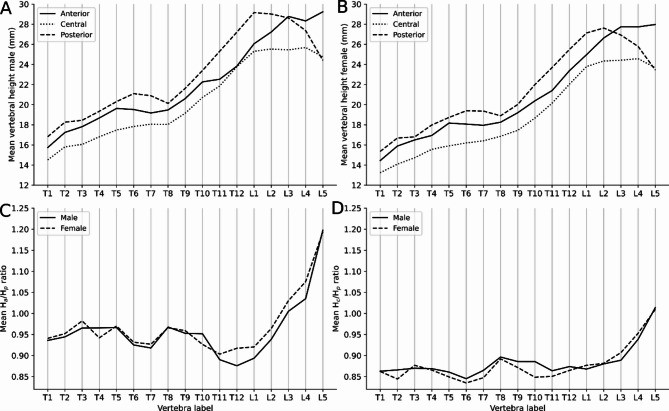
Mean Ha, Hp and Hc highlighting a decrease of the posterior height at L3 in males (**A**) and before L3 in females (**B**). Comparison of the Anterior Wedge Indices demonstrate higher results T12–L4 in females compared to males (**C**). Biconcavity indices of males and females are comparable (**D**).

In the kyphotic thoracic spine of both sexes, the mean H_a_ was lower than the mean H_p_ of the same vertebra (Fig. 4).

In the lordotic lower lumbar spine the posterior heights of the lumbar spine showed a decrease in craniocaudal direction with higher H_a_ than H_p_, relating to moderate dorsal wedge-shaped vertebrae (Fig. 4A,B). The Anterior Wedge Index (H_a_/H_p_) in T12 (*p* < 0.001), L1 (*p* < 0.02), L2 (*p* < 0.02), L3 (*p* < 0.03) and L4 (*p* = 0.001) was higher in females, demonstrating a stronger, dorsal wedge-shape (Table 4; Fig. 4C). The biconcavity index (H_c_/H_p_) in males and females was comparable with a significant difference only in T10, p-value < 0.001 (Table 4; Fig. 4D).

**Table 4:** Mean values of anterior wedge index (H_a_/H_p_) and biconcavity index (H_c_/H_p_) for males and females. P-values show significant sex-associated differences for H_a_/H_p_ at T12, L3 and L4 as well as for H_c_/H_p_ at T10.

	Anterior wedge index (Ha/Hp)			Biconcavity index (Hc/Hp)		
	Male	Female	P-value	Male	Female	P-value
T1	0.93	0.94	0.77	0.86	0.86	0.91
T2	0.94	0.96	0.70	0.87	0.84	0.10
T3	0.96	0.99	0.26	0.87	0.88	0.67
T4	0.96	0.94	0.14	0.87	0.87	0.85
T5	0.97	0.98	0.87	0.86	0.85	0.30
T6	0.92	0.93	0.70	0.85	0.83	0.51
T7	0.92	0.92	0.69	0.86	0.85	0.24
T8	0.97	0.97	0.54	0.90	0.89	0.58
T9	0.94	0.96	0.80	0.89	0.87	0.20
T10	0.94	0.94	0.08	0.89	0.85	< 0.001
T11	0.88	0.91	0.37	0.86	0.85	0.18
T12	0.87	0.92	< 0.001	0.87	0.86	0.20
L1	0.89	0.93	0.02	0.87	0.88	0.26
L2	0.94	0.97	0.02	0.88	0.88	0.91
L3	1.00	1.04	0.03	0.89	0.91	0.07
L4	1.03	1.08	0.001	0.94	0.95	0.11
L5	1.20	1.19	0.71	1.01	1.01	0.68

### Differences in sex-adapted mixed model regression

**Fig. 5:**
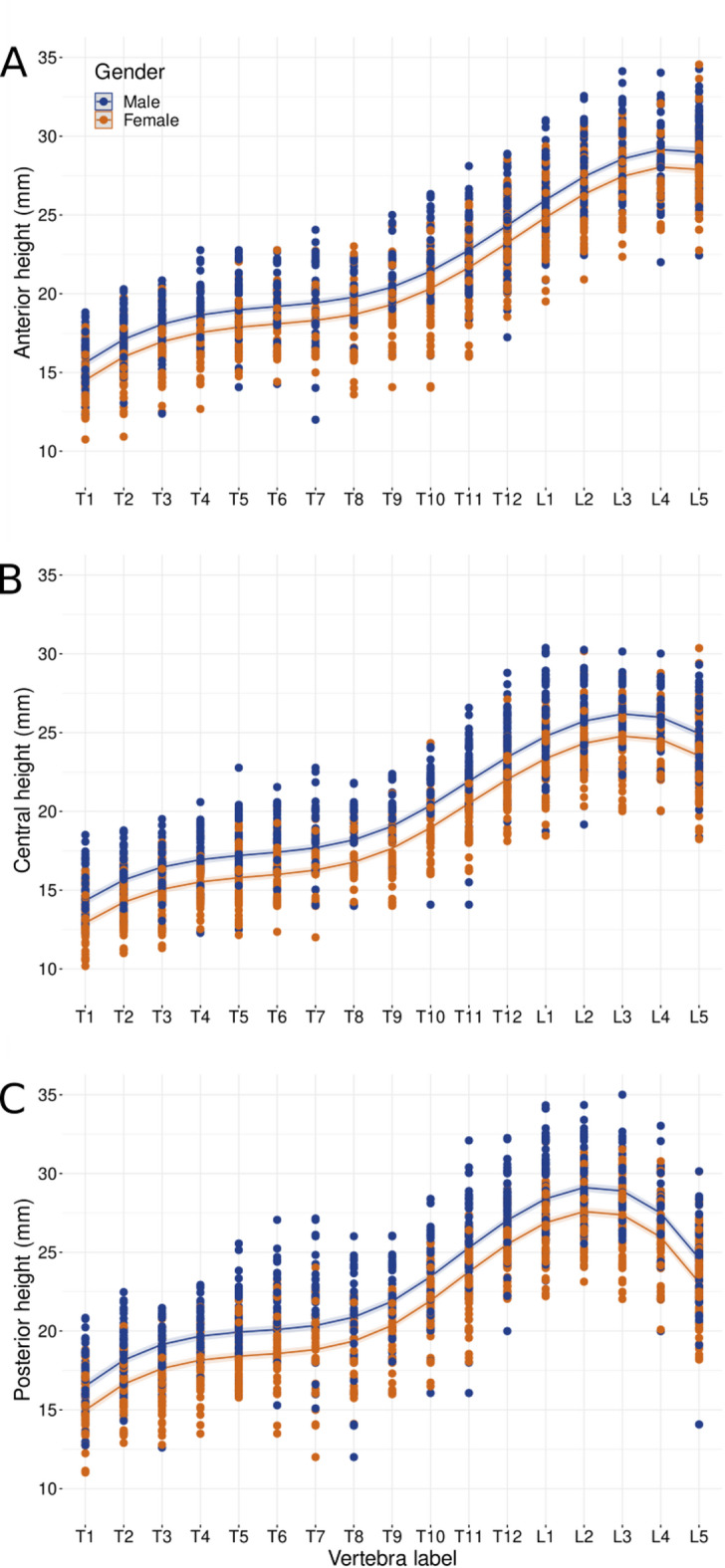
Cubic spline mixed effects regression model with random effects to model the S-shaped vertebral height distribution.

The cubic spline mixed effects regression model with random effects confirmed the S-shaped vertebral height distribution (Fig. 5) resulting in the following equation:

$$\begin{aligned}\: & Vertebral\:height{\:}_{ij} \\ & ={\beta\:}_{0}+{b}_{0i}+{\beta\:}_{1}ve{r}_{ij}+{b}_{1i}ve{r}_{ij}+{\beta\:}_{2}ve{r}_{ij}^{2}+{\beta\:}_{3}ve{r}_{ij}^{3}+{\beta\:}_{4}{\left(ve{r}_{ij}-9\right)}_{+}^{3} \\ &+{\beta\:}_{5}{\left(ve{r}_{ij}-13\right)}_{+}^{3}+{\beta\:}_{11}I\left(male\right)+{\epsilon\:}_{ij}\end{aligned}$$

Compared to standardized residuals from ordinary least squares regression (Fig. S2A) an improved fit was found by including random effects into the model (Fig. S2B). This was confirmed in the Akaike information criterion (AIC) and Bayesian Information Criterion (BIC) when comparing the different models (Table S3). To demonstrate the non-linearity in the vertebral height distribution curve, a cubic spline regression model was tested with varying number and positions of knots with identification of knots at T9 and L1. The resulting cubic spline regression model with random effects fitted the S-shaped vertebral height distribution in a sex-independent manner. Supplement Table S4, S5, S6 show the estimated parameters of the mixed effects and Supplement Table S7, S8, and S9 of the sex-stratified cubic spline regression model for H_a_, H_c_, and H_p_.

### Sex-independent vertebral height ratios in the Pseudo-Jacobian matrices

**Fig. 6:**
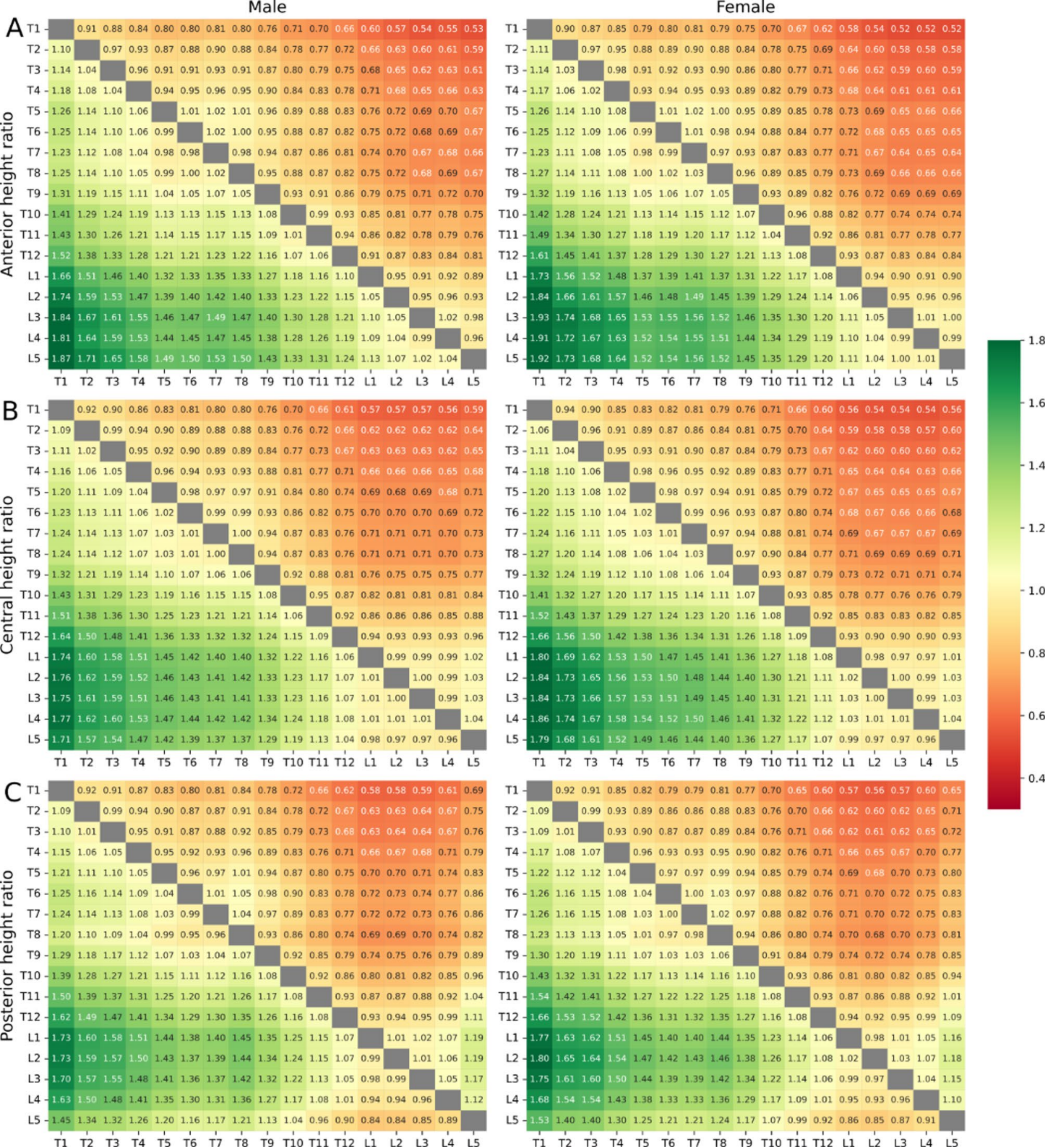
Pseudo-Jacobian Matrix for male on the left and female on the right. (**A**–**C**) show Ha, Hp and Hc ratios to every other vertebra in the spine.

The algebraic Pseudo-Jacobian Matrix for males and females demonstrates the mean H_a_, H_c_, H_p_ – ratios of adjacent and distant vertebral bodies (Fig. 6A–C).

The vertebral height ratios did not show any significant difference between male and female (Table 5).

**Table 5:** Statistical significance tests comparing differences in H_a,_ H_p_ and H_c_ ratios of males and females in the study cohort.

	Anterior height ratio		Central height ratio		Posterior height ratio	
	W-statistic	P-value	W-statistic	P-value	W-statistic	P-value
T1	0.32	0.75	0.16	0.88	0.29	0.77
T2	0.26	0.80	0.49	0.63	0.27	0.79
T3	0.19	0.85	0.07	0.94	0.34	0.74
T4	0.49	0.63	− 0.06	0.95	0.002	0.99
T5	0.19	0.85	0.19	0.85	0.13	0.90
T6	0.19	0.85	0.22	0.83	0.18	0.86
T7	0.03	0.98	0.22	0.83	0.05	0.96
T8	0.01	0.99	− 0.19	0.85	− 0.16	0.88
T9	0.09	0.93	0.18	0.86	0.07	0.94
T10	0.35	0.73	0.33	0.74	− 0.18	0.86
T11	− 0.19	0.85	0.002	0.99	− 0.11	0.91
T12	− 0.65	0.52	− 0.06	0.95	− 0.11	0.91
L1	− 0.31	0.76	− 0.27	0.79	− 0.01	0.99
L2	− 0.61	0.55	− 0.48	0.64	− 0.37	0.71
L3	− 0.40	0.69	− 0.57	0.57	− 0.18	0.86
L4	− 0.63	0.54	− 0.53	0.60	− 0.21	0.83
L5	− 0.29	0.77	− 0.52	0.61	− 0.52	0.61

The Pseudo-Jacobian Matrices of the mean H_a_ and H_c_ – ratios demonstrate a sex-independent mean ratio close to one in T5–T8 (male 0.98–1.03; female 0.99–1.05) and in the lower lumbar spine, correspondingly to the shown height plateaus (Fig. S1, Fig. 6A,B). The highest height ratios were found in T9–L1 (male 1.01–1.10; female 1.03–1.09), relating to the strongest height increase from one vertebra to the other in the craniocaudal direction. Correspondingly, the three-times-three algebraic Pseudo-Jacobian mean height matrix of the regions T1–T9, T10–L1, and L2–L5 demonstrate minor sex differences especially in the lower lumbar spine (Fig. S3), however with no statistically significant differences between males and females (Table 6).

**Table 6:** Region-based statistical significance test comparing differences in H_a,_ H_p_ and H_c_ ratios of males and females in the study cohort.

	Anterior height ratios		Central heights ratios		Posterior heights ratios	
	W-statistic	P-value	W-statistic	P-value	W-statistic	P-value
Mean T1 to T9	0.12	0.91	0.09	0.93	0.08	0.94
Mean T10 to L1	− 0.04	0.97	0.03	0.98	− 0.01	0.99
Mean L2 to L5	− 0.14	0.90	− 0.17	0.88	0.10	0.93

## Discussion

Our analyses of 3117 vertebrae showed a sex-independent S-shaped vertebral height distribution with a height plateau from T5 to T9 and L2 to L5. Vertebral height differences in males and females were statistically significant. The cubic spline mixed effects regression model including random effects achieved the best fit for description of the vertebral height distribution. A consistently lower intercept was observed in the female regression model. The algebraic Pseudo-Jacobian Matrix confirmed two sex-independent height plateaus. Female vertebrae L2–L4 showed a discrete more posterior wedge shape, resulting in a more lordotic alignment with a mildly higher Anterior Wedge Index.

### Vertebral morphology and sex-based differences

Several population-based studies have provided a normal variation of vertebral heights along the spinal column by assessing vertebral heights in medical imaging^34–37^. In a radiography-based study, the anterior, central and posterior height distribution in males and females from T4 to L5 in a Chinese study cohort showed comparable results to those observed in the current study^33^. Similar to our study, the vertebral height increased with each consecutive vertebra with a plateau at the lumbar segment. Female vertebral heights also showed overall lower values than male vertebrae. This is consistent with American or British population-based study results^16,24,38,39^. However, absolute vertebral heights from our cohort were the lowest compared to the UK, American and Chinese populations^16,24,38,39^. A possible explanation could be a lower anatomical precision when measuring the heights in radiographs than CT, potentially leading to higher measurement values caused by a superimposition in radiographs. In addition, CT images show higher anatomical precision with correspondingly improved measurement accuracy which is underlined by comparison of our study results with the anatomical vertebral body shape study from Masharawi et al.^23^. Analysis of dissected vertebrae from male and female individuals showed vertebral body heights close to our CT-based, automated measurements^23^. Moreover, sex-associated differences with significantly higher values in males were demonstrated^23^. The findings of Masharawis et al. also aligned with the distributions of anterior and posterior height as illustrated in our Fig. 5 with peak kyphosis and anterior wedging in T7; as well as peak lordosis corresponding with posterior wedging in L5^23^. Sevinc et al. evaluated morphometric features of the lumbar spine based on height measurements in MRI^17^. Consistent with our findings, the study demonstrates more wedge-shaped vertebrae with a higher anterior wedge index in the mid and lower lumbar spine in female participants. However, this was not found to be statistically significant^17^.

Linear and multiple regression analyses on the spine have been shown to be useful methods to evaluate body height in either post-mortem or post-vertebral fracture patients^1,6,14,40,41^. An analysis of various existing regression models by Flanders et al. predicting vertebral heights in an Australian cohort demonstrated the most accurate results using pooled-sex multiple regression models from a vertebral dimension study of middle-aged Finns^1,5^. Here, the lowest prediction error was achieved at L4 by multiple linear regression models with a sex-analogous trend, but with sex-dependent accuracy^5^. From our knowledge a cubic spline mixed effects regression model including random effects comparable to our study has not been published before. In our study cohort from Germany, these newly developed models showed a superior fit than linear models or models without random effects. Corresponding to previous studies, we showed an improved fit in pooled-sex models rather than sex-stratified models^5^. Sex-associated differences in the lower lumbar spine were not statistically significant. However, our results demonstrate significant sex-differences in vertebral heights consistent with previous studies^42,43^. Since previous models only yielded a fit to a specific spinal region or vertebral segment, our approach is expected to provide an improved, holistic algebraic method with an advanced, sex-adapted fit in thoracolumbar vertebrae^1,5,9,44,45^. Using linear regression models to analyze vertebral height distribution, our results demonstrate the best fit dividing the vertebrae into T1–T9, T10–L1 and L2–L5, rather than two linear regression models based on the anatomical regions T1–T12 and L1–L5 as published by Nagesh et al.^9^. A reasonable explanation is that the S-shaped height distribution naturally relies on two graphical plateaus (T5–T9 and L2–L5) with a steep transition zone (T10–L1), thereby resulting in a steeper slope. Hence, a linear algebraic approach to analyze this distribution demonstrates the highest accuracy by adaptation to these three regions.

To our knowledge, the algebraic evaluation of inter-vertebral height ratios with a Pseudo-Jacobian Matrix have never been done. However, the singular inter-vertebral height ratio distribution between neighboring vertebrae has shown comparable results in previous studies. Zebaze Djoumessi et al. demonstrated comparable study results of neighboring inter-vertebral height ratios reflecting the inflection points of the spine with the smallest ratio range at the mid-thoracic and thoracolumbar junction^46^.

### Clinical Implementations, AI and Future Directions

Prior studies have shown that opportunistic CT-based fracture prediction models can effectively assess vertebral integrity and predict fracture risk^47^. Similarly, AI-based estimation of bone mineral density (BMD) and osteoporosis in routine CT examinations may have the potential to improve osteoporosis management through early disease detection^48^. By integrating the Pseudo-Jacobian matrix approach, our study provides a robust framework for intra-individual vertebral height comparisons. This approach could refine current AI-based osteoporosis screening models for osteoporosis risk stratification and fracture prediction, facilitating early vertebral compression fracture detection and personalized treatment strategies, particularly in high-risk populations.

Our findings further contribute to the growing field of AI-assisted vertebral morphometry by providing updated, sex-specific vertebral height reference values. Given that vertebral morphology directly influences spinal load distribution, our updated vertebral height measurements could serve as critical inputs for biomechanical models, particularly in finite element modeling (FEM) for spinal load assessment and orthopedic implant design^49,50^. Additionally, the application of CT-based morphometric sex estimation models in forensic anthropology has demonstrated improved accuracy over traditional radiographic methods^51,52^. Building on this, our AI-driven CT segmentation approach, with a standardized measurement method, may also offer higher reproducibility than manual morphometry, supporting future clinical and forensic applications.

In the future, AI-driven imaging assessment and biobanks may further enhance fracture risk assessment, early diagnosis, and personalized treatment strategies. Imaging biobanks in combination with AI may further facilitate automated bone mineral density evaluation, improving risk stratification, reducing diagnostic variability, improving osteoporosis classification accuracy by standardization^48,53^. Imaging biobanks facilitate large-scale AI-driven risk assessment but require improved data standardization and interoperability^54^. Moreover, recent causal analyses indicate that while bone health and cardiovascular disease share common risk factors, no direct causal link has been established, highlighting the potential for integrated imaging approaches to assess both osteoporosis and cardiovascular health within precision medicine frameworks^55^. As a future direction, research needs to explore how AI and imaging biobanks can be improved by sex-specific vertebral height models, as demonstrated in our study, to refine osteoporosis risk stratification, as well as advance personalized diagnostic and targeted treatment strategies.

This study was conducted on a visually anatomically healthy cohort which could be subject to observer bias and selection bias. The mean age of the study cohort was 32.3 years (20–54 years). This may not be fully representative of vertebrae in other age groups, especially due to age-dependent changes in the spine^17^. An age-stratified analysis was not feasible due to the cohort selection criteria and correspondingly limited age variability. BMI, body composition, medication and further clinical information, which may have an effect on vertebral height, were not taken into account^56^. Measuring the vertebral heights precisely is dependent on the pre-trained model with corresponding CT acquisition parameters, imaging reconstruction and overall image quality. Automated segmentations are subject to error, which may lead to inaccurate anatomical measurements or misinterpretation of results. All segmentations were manually reviewed and missegmentations excluded. Quantitative evaluation of the TotalSegmentator tool on our dataset was not assessed, and therefore its performance metrics on this specific population remain undetermined.

## Conclusion

In conclusion, the Pseudo-Jacobian nomogram developed in this study provides sex-specific reference values for vertebra height for each segment to improve orthopedic and forensic vertebral height assessment. Such data allows for the prediction of individual vertebral height and may facilitate compression fracture detection in future studies.

## Electronic supplementary material

Below is the link to the electronic supplementary material.

Supplementary Material 1

## Data Availability

The datasets analysed during the current study are not publicly available due to ethical restrictions but are available from the corresponding author on reasonable request. Data are located in controlled access data storage at University Hospital Heidelberg, Diagnostic and Interventional Radiology Research Department. Access may be subject to institutional policies, ethical approval, and data-sharing agreements.

## Code availability

Due to institutional restrictions, the algorithms generated during the current study are only available from the corresponding author on reasonable request. Algorithms are located in controlled access data storage at University Hospital Heidelberg, Diagnostic and Interventional Radiology Research Department. Access may be subject to institutional policies, ethical approval, and data-sharing agreements.
