# Supplementary material for: AI-based CT assessment of 3117 vertebrae reveals significant sex-specific vertebral height differences
Source: Sci Rep. 2025 Jul 1;15:20756. doi: 10.1038/s41598-025-05091-0 (PMC12218285; doi:10.1038/s41598-025-05091-0)

Supplement

Table S1: ICC between two readers and automated measurements showing a comparable measurement accuracy

	ICC (2, k)	p-value	95% CI of ICC Population values
R1 vs. R2	0.98	<.001	0.96 - 0.99
R1 vs. Algorithm	0.94	0.001	0.64 - 0.98
R2 vs. Algorithm	0.96	<.001	0.90 - 0.98

Figure S1: Boxplot of H_a (A) H_c (B) and H_p (C) showing an S-shaped distribution of vertebral heights

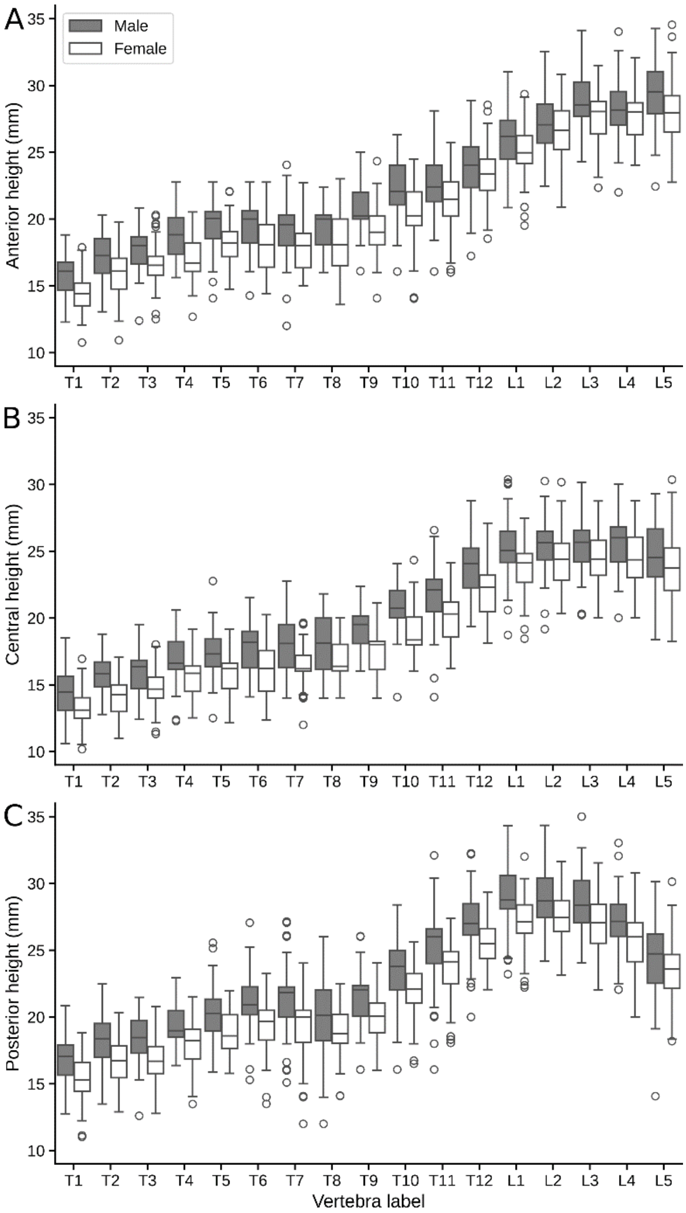

Table S2: Mean H_a, H_p, H_c and SD of male and female

	Anterior height (mm) Mean ± SD		Central height (mm) Mean ± SD		Posterior height (mm) Mean ± SD	
	Male	Female	Male	Female	Male	Female
T1	15.74 ± 1.56	14.45 ± 1.45	14.51 ± 1.66	13.24 ± 1.44	16.82 ± 1.85	15.35 ± 1.65
T2	17.24 ± 1.64	15.89 ± 1.58	15.81 ± 1.50	14.09 ± 1.48	18.26 ± 1.91	16.69 ± 1.55
T3	17.82 ± 1.51	16.50 ± 1.54	16.05 ± 1.48	14.73 ± 1.52	18.45 ± 1.69	16.79 ± 1.63
T4	18.68 ± 1.68	16.94 ± 1.44	16.81 ± 1.63	15.57 ± 1.29	19.35 ± 1.55	17.99 ± 1.60
T5	19.63 ± 1.78	18.17 ± 1.61	17.48 ± 1.66	15.91 ± 1.47	20.31 ± 1.96	18.73 ± 1.58
T6	19.52 ± 1.88	18.07 ± 1.63	17.83 ± 1.81	16.20 ± 1.61	21.10 ± 2.11	19.40 ± 1.95
T7	19.17 ± 2.28	17.95 ± 1.60	18.06 ± 1.87	16.41 ± 1.43	20.89 ± 2.66	19.37 ± 2.32
T8	19.48 ± 1.57	18.26 ± 1.91	18.04 ± 1.86	16.86 ± 1.47	20.13 ± 2.79	18.90 ± 1.92
T9	20.61 ± 1.58	19.20 ± 1.76	19.16 ± 1.60	17.45 ± 1.56	21.63 ± 2.05	20.02 ± 1.73
T10	22.27 ± 2.02	20.39 ± 2.07	20.72 ± 1.65	18.67 ± 1.77	23.40 ± 2.28	22.01 ± 1.94
T11	22.53 ± 1.94	21.41 ± 2.00	21.86 ± 2.06	20.16 ± 1.62	25.31 ± 2.62	23.70 ± 1.99
T12	23.83 ± 2.31	23.37 ± 1.97	23.78 ± 1.93	22.03 ± 1.69	27.21 ± 2.15	25.48 ± 1.68
L1	26.06 ± 2.05	24.97 ± 1.82	25.31 ± 2.22	23.79 ± 1.73	29.16 ± 2.31	27.13 ± 1.78
L2	27.23 ± 2.22	26.63 ± 2.00	25.54 ± 2.01	24.35 ± 1.87	29.01 ± 2.17	27.62 ± 1.84
L3	28.78 ± 2.07	27.74 ± 1.94	25.45 ±1.93	24.42 ± 2.01	28.63 ± 2.10	26.93 ± 2.16
L4	28.33 ± 2.23	27.74 ± 1.72	25.69 ±1.93	24.58 ± 1.95	27.37 ± 2.21	25.79 ± 2.32
L5	29.23 ± 2.31	27.98 ± 2.13	24.75 ±2.19	23.67 ± 2.37	24.41 ± 2.56	23.44 ± 2.36

Figure S2: Standardized residuals from ordinary least squares regression (A) and regression with random effects (B) showing improved fit by including random effects to the model.

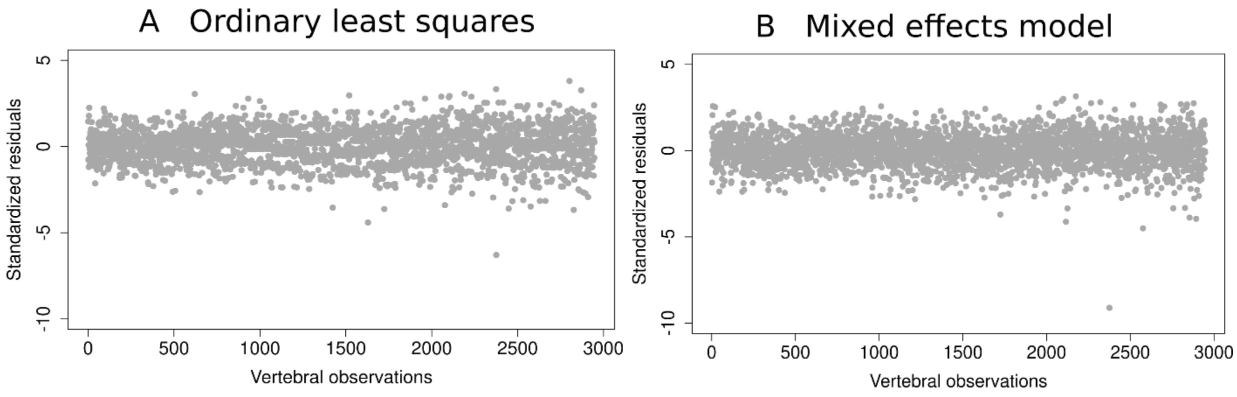

Table S3: AIC and BIC values for the different regression models with improved fit when including random effects into the model

	**Anterior height**		**Central height**		**Posterior height**	
	AIC	BIC	AIC	BIC	AIC	BIC
**Ordinary least square regression (no random effects)**	12966.36	12984.3	12779.88	12797.83	14210.86	14228.81
**Mixed effects linear regression model**	12404.54	12428.48	11691.97	11715.89	13744.9	13768.82
**Ordinary least square regression with cubic splines (no random effects)**	12338.1	12379.97	12143.18	12185.05	12987.14	13029.01
**Mixed effects model with cubic spline regression**	11462.6	11510.45	10413.53	10461.38	11926.48	11974.33

Table S4: Estimated parameters from cubic spline regression models for anterior heights

	**Mixed effects model with cubic spline regression**		
	Estimates	Confidence Interval	p-value
**Intercept**	14.52	14.20 - 14.83	<.001
**β1**	4.74	4.18 - 5.30	<.001
**β2**	2.08	1.71 - 2.45	<.001
**β3**	8.61	8.19 - 9.03	<.001
**β4**	14.55	14.10 - 14.99	<.001
**β5**	13.37	13.02 - 13.71	<.001
**b11 I(Male)**	1.11	0.80 - 1.41	<.001
**σ^2**	2.43	-	-
**Marginal R^2**	0.82	-	-
**Conditional R^2**	0.89	-	-

Table S5: Estimated parameters from cubic spline regression models for central heights

	**Mixed effects model with cubic spline regression**		
	Estimates	Confidence Interval	p-value
**Intercept**	12.93	12.63 - 13.22	<.001
**β1**	4.27	3.82 - 4.73	<.001
**β2**	1.16	0.85 - 1.46	<.001
**β3**	9.76	9.42 - 10.10	<.001
**β4**	13.60	13.23 - 13.97	<.001
**β5**	10.60	10.32 - 10.89	<.001

**b11 I(Male)**	1.41	1.08 - 1.74	<.001
**σ^2**	1.61	-	-
**Marginal R^2**	0.82	-	-
**Conditional R^2**	0.91	-	-

Table S6: Estimated parameters from cubic spline regression models for posterior heights

	**Mixed effects model with cubic spline regression**		
	Estimates	Confidence Interval	p-value
**Intercept**	14.99	14.64 - 15.33	<.001
**β1**	5.43	4.82 - 6.03	<.001
**β2**	1.03	0.63 - 1.43	<.001
**β3**	11.46	11.01 - 11.92	<.001
**β4**	15.51	15.03 - 15.99	<.001
**β5**	8.10	7.73 - 8.47	<.001
**b11 I(Male)**	1.52	1.18 - 1.86	<.001
**σ^2**	2.82	-	-
**Marginal R^2**	0.78	-	-
**Conditional R^2**	0.86	-	-

Table S7: Sex-stratified cubic spline regression estimates for anterior heights

	**Male**			**Female**		
	Estimates	Confidence interval	p-value	Estimates	Confidence interval	p-value
**Intercept, β_0**	15.62	15.20 - 16.03	<.001	14.46	14.11 - 14.82	<.001
**β_1**	4.98	4.20 - 5.76	<.001	4.87	4.17 - 5.57	<.001
**β_2**	1.49	0.92 - 2.07	<.001	1.14	0.62 - 1.66	<.001
**β_3**	10.41	9.78 - 11.04	<.001	10.77	10.20 - 11.33	<.001
**β_4**	13.94	13.32 - 14.57	<.001	14.42	13.88 - 14.97	<.001
**β_5**	13.54	13.01 - 14.06	<.001	13.31	12.86 - 13.77	<.001
**σ^2**	2.57	-	-	2.29	-	-
**Marginal R^2**	0.80	-	-	0.84	-	-
**Conditional R^2**	0.88	-	-	0.89	-	-

Table S8: Sex-stratified cubic spline regression estimates for central heights

	**Male**			**Female**		
	Estimates	Confidence interval	p-value	Estimates	Confidence interval	p-value
**Intercept, β_0**	14.26	13.88 - 14.63	<.001	12.96	12.63 - 13.29	<.001
**β_1**	4.48	3.84 - 5.12	<.001	4.32	3.76 - 4.88	<.001
**β_2**	0.37	-0.10 - 0.84	0.12	0.49	0.07 - 0.91	0.02
**β_3**	12.33	11.81 - 12.85	<.001	11.56	11.10 - 12.01	<.001
**β_4**	11.81	11.29 - 12.32	<.001	12.46	12.02 - 12.90	<.001
**β_5**	10.73	10.29 - 11.16	<.001	10.76	10.39 - 11.13	<.001
**σ^2**	1.72	-	-	1.48	-	-

**Marginal R^2**	0.80	-	-	0.83	-	-
**Conditional R^2**	0.91	-	-	0.92	-	-

Table S9: Sex-stratified cubic spline regression estimates for posterior heights

	**Male**			**Female**		
	Estimates	Confidence interval	p-value	Estimates	Confidence interval	p-value
**β_0**	16.40	15.94 - 16.86	<.001	15.04	14.66 - 15.42	<.001
**β_1**	5.82	4.96 - 6.68	<.001	5.32	4.59 - 6.05	<.001
**β_2**	-0.27	-0.90 - 0.37	0.41	0.33	-0.21 - 0.87	0.24
**β_3**	14.55	13.86 - 15.25	<.001	13.81	13.23 - 14.40	<.001
**β_4**	12.80	12.80 - 13.49	<.001	12.46	11.89 - 13.03	<.001
**β_5**	8.10	7.52 - 8.68	<.001	8.37	7.90 - 8.85	<.001
**σ^2**	3.14	-	-	2.50	-	-
**Marginal R^2**	0.75	-	-	0.80	-	-
**Conditional R^2**	0.85	-	-	0.87	-	-

Figure S3: Sex differences of three vertebral regions defined by mixed model regression analysis in T1-T9, T10-L1, L2-L5 highlighting sex associated tendencies in the lower lumbar spine

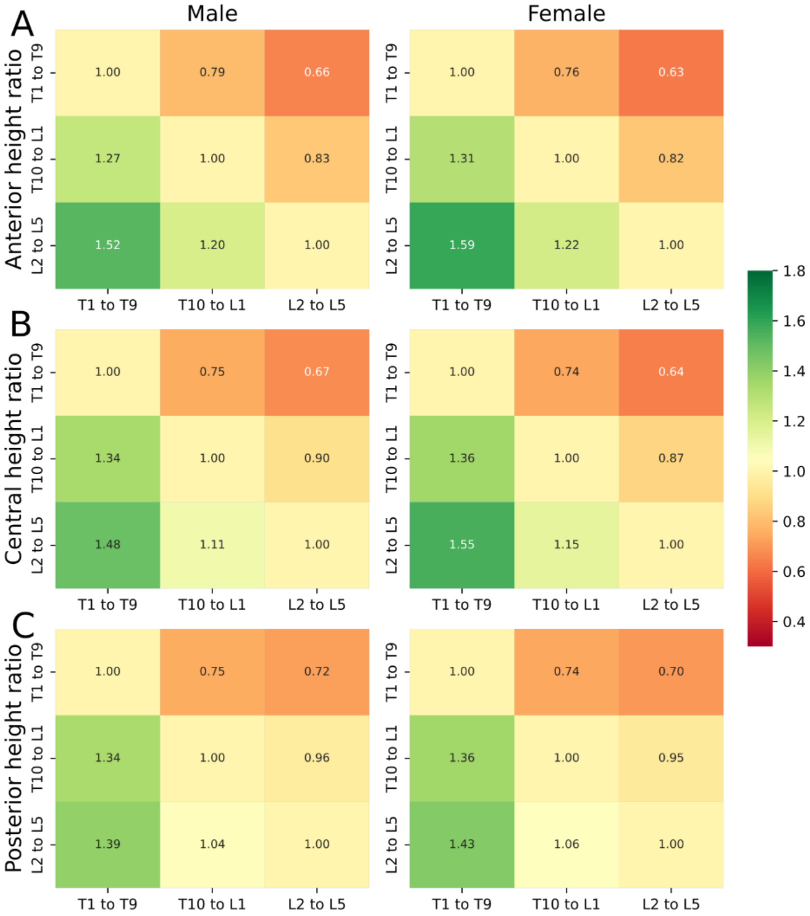

Supplement: Supplementary file 1 — Supplementary Material 1 [file 41598_2025_5091_MOESM1_ESM.pdf]
